# Formulation Optimization, Quality Characterization, and Flavor Profiling of Cookies Enriched with Ultrafine Dark Tea Powder

**DOI:** 10.3390/foods15050880

**Published:** 2026-03-04

**Authors:** Xiaoping Huang, Ang Li, Xiao Zhou, Peiran Li, Jiaojunnan Huang, Lin Shao, Yaqiong Pei

**Affiliations:** 1 College of Food Science and Technology, Wuhan Business University, Wuhan 430056, China; liangtmu@163.com (A.L.); 15071532667@163.com (X.Z.); 15633972091@163.com (P.L.); 18972558477@163.com (J.H.); 2 College of Animal Science & Technology, College of Veterinary Medicine, Huazhong Agricultural University, Wuhan 430070, China; shaolin@new-pets.cn

**Keywords:** cookie, ultrafine dark tea powder, formulation optimization, quality characterization, flavor profiling

## Abstract

Ultrafine dark tea powder (UDTP) was prepared by superfine grinding and sieving through a 200-mesh sieve, and incorporated into cookies to improve their textural properties, sensory acceptability and flavor characteristics. Through single-factor experiments and orthogonal testing, the optimal formulation was determined. The quality of cookies was evaluated by texture analysis, sensory evaluation, electronic nose (E-nose), and gas chromatography-mass spectrometry (GC-MS). Results showed that cookies supplemented with 4 g UDTP per 80 g butter exhibited significantly lower hardness and comparable fracturability, along with higher sensory scores in texture, odor and taste compared to basic butter cookies. E-nose and GC-MS analyses revealed that UDTP enrichment promoted the formation of desirable volatile compounds, particularly aldehydes, ketones, and heterocyclic compounds, which contributed to floral, fruity, roasted nutty, and caramel aromas. This study demonstrates that UDTP can effectively improve both the textural and flavor properties of cookies, providing a viable approach for developing tea-fortified baked products with enriched sensory profiles.

## 1. Introduction

Cookies are widely consumed globally due to their convenience, long shelf life, affordability, and ease of preparation. In many developed countries, they are also considered an appropriate medium for food enrichment [1,2]. Despite the maturity of cookie formulation and production techniques, as well as their widespread consumer acceptance, the cookie industry faces significant challenges, such as the prevalence of homogeneous product offerings. Consequently, nutritional fortification and flavor innovation have emerged as crucial research and development priorities in the baking industry.

Ultrafine grinding technology, a novel method developed in China and Japan during the 1990s, utilizes mechanical or fluid power to reduce materials into powders with particle sizes at the micron, submicron, and nanometer levels [3,4,5]. This advanced technology achieves a tea cell disruption rate exceeding 95%, effectively preserving all active components of tea, including polyphenols, proteins, and dietary fiber, while maintaining its original color, nutritional value, and functional properties [6]. Compared with conventional tea powder, ultrafine tea powder exhibits higher specific surface area, better dispersion uniformity, and enhanced release of bioactive compounds, which can significantly improve the texture, flavor, and functional properties of food products [6,7,8,9]. As a result, ultrafine tea power has been increasingly applied in the production and processing of foods such as cookies, cakes, bread, and noodles to enhance flavor quality and inhibit oxidative deterioration [7,8,9].

Dark tea, a type of post-fermented tea with distinctive sensory characteristics, is produced through the specialized fermentation of microorganisms [10,11]. It has become the second largest tea category in China, following green tea [12]. Dark tea contains numerous bioactive compounds, including tea polyphenols, thea brownin and tea polysaccharides, which have been reported to confer various health benefits [13,14,15,16]. Thus, dark tea has garnered increasing consumer interest, particularly in China and Southeast Asia.

Previous studies have primarily focused on the functional properties of dark tea extracts or infusions, but research on its application in solid food products, especially using ultrafine grinding technology, remains relatively limited. The incorporation of whole dark tea powder into baked goods could provide a natural way to enhance flavor and potentially deliver functional benefits. However, systematic studies on the optimization of formulations and the impact on product quality are limited.

Therefore, this study aimed to develop UDTP-enriched cookies through formulation optimization, and comprehensively evaluate their textural properties, sensory characteristics, and volatile flavor compounds. The research provides a reference for developing distinctive tea-based snack foods and improving the utilization efficiency of tea resources.

## 2. Materials and Methods

### 2.1. Material and Chemical Reagents

Dark tea was purchased from Anhua Shuanglongxi Tea Industry Co., Ltd., Yiyang, China. The dried dark tea was ground using a Yun Bang model YB-1500A multifunctional grinder (Yongkang Sufeng Industry and Trade Co., Ltd., Yongkang, Zhejiang, China) operating at 220 V with a power of 3500 W and a motor speed of 25,000 rpm. The ground power was then sieved through a 200-mesh sieve (theoretical particle size ≤ 74 μm) to obtain UDTP [17]. Low-gluten wheat flour (LGWF) was purchased from Nanshun Food Co., Ltd., Shekou, Changzhou, China. Icing sugar was purchased from Dongguan Yibao Import and Export Co., Ltd., Guangdong, China. Salt, butter, almond powder and eggs were purchased from a local supermarket in Hubei, China. Other chemical reagents were obtained from Sigma-Aldrich Co., Ltd. (St. Louis, MO, USA) and were of analytical grade.

### 2.2. Morphological Characteristics of UDTP

The morphology of UDTP was observed using a scanning electron microscope (SEM; TM4000, Tokyo, Japan) at an accelerating voltage of 5 kV. UDTP samples were freeze-dried for 24 h using an FD5-3T freeze-dryer (Beijing Jin Ximeng Instrument Co., Ltd., Beijing, China) to remove moisture, then mounted on a metal stage, sputter-coated with gold, and observed at random. For comparison, unsieved dark tea power samples were also photographed and recorded.

### 2.3. Cookie Preparation

#### 2.3.1. Basic Formulation of Cookies

The basic cookie formula was prepared according to the method described by Wang et al. with minor modifications [3]. The basic formulation (without UDTP) of cookies is listed in Table 1. The cookies prepared without UDTP served as the control group and are referred to as basic butter cookies.

#### 2.3.2. Preparation of Cookies

Cookies were prepared according to the following steps: First, butter was softened. Icing sugar was then blended with the butter and beaten until pale in color. Next, UDTP, LGWF and other ingredients were added to the mixer and mixed at medium speed until no dry flour was visible. The cookie dough was stored at −20 °C for 60 min to firm up, then removed and cut into pieces measuring 20 mm × 20 mm × 8 mm. The dough pieces were baked in an oven at 160 °C for 18 min until the cookie surface turned golden brown. Finally, the baked cookies were cooled to room temperature, packaged in light-proof materials, and stored at ambient temperature.

#### 2.3.3. Single-Factor Experiment

Four factors, namely UDTP, LGWF, salt, and icing sugar, were selected to evaluate their effects on the quality characteristics of cookies (Table 2). In each single-factor experiment, one specific factor was varied while the others were kept constant. The optimal parameters identified from the previous single-factor experiment were used as the basis for subsequent single-factor experiments. Each single-factor experiment was conducted in triplicate.

#### 2.3.4. Orthogonal Test

Based on the results of the single-factor experiments, four factors (A: LGWF; B: Salt; C: Icing sugar; D: UDTP) were identified and their appropriate ranges were determined. A four-factor, three-level orthogonal experiment was conducted to further optimize the formulation (Table 3).

### 2.4. Sensory Evaluation

Sensory evaluation of the cookies was followed by an assessment of the general quality of the biscuits (GB/T 20980-2021) [18]. The evaluation was conducted in a standardized sensory laboratory at Wuhan Business University, equipped with individual booths and controlled lighting and temperature (22 ± 2 °C). The sensory panel consisted of 15 trained assessors aged 20–26 (students at the College of Food Science and Technology, Wuhan Business University), who had completed a 30 h training course in sensory analysis and had prior experience in evaluating baked products. Each session evaluated up to six samples, presented in random order with different codes. For each group of sensory scores, the highest and lowest scores were excluded, and the mean of the remaining scores was calculated as the final result. Cookie samples were evaluated using a 100-point scale, including color (0–20 points), appearance (0–20 points), odor and taste (0–35 points) and texture (0–25 points).

### 2.5. Texture Analysis

Texture analysis was performed using a texture analyzer (TA-XT Plus, SMS, Godalming, Surrey, UK) [19,20]. The test parameters were set as follows: P2 for the probe, 5 g of trigger force, and 3 mm of compression distance. The speed was 1 mm/s pre-test, 1 mm/s during the test, and 1 mm/s post-test. The measured parameters were hardness and fracturability. Hardness is defined as the maximum force required to break a cookie and fracturability is given by the load force of the first significant peak. Data acquisition and analysis were performed using Exponent software, version 6.1.23.0 (Stable Micro Systems Ltd., Godalming, Surrey, UK). Each sample was tested at least three times.

### 2.6. Electronic Nose Analysis

The flavor compounds of cookies were detected by an E-nose instrument (AIRSENSE-PEN 3, Beijing Yingsheng Hengtai Technology Co., Ltd., Beijing, China) [21]. Approximately 10 g of cookies were added to a 50 mL headspace vial and enriched at 25 °C for 30 min. The detection method was adapted from the research by Song et al., with slight modifications [22]. The E-nose operating conditions were set as follows: inlet preparation time: 80 s, flow rate: 400 mL/min, detection time: 60 s, wash time 80 s.

The E-nose system consisted of an array of 10 metal oxide sensors, each sensitive to specific volatile compounds, as detailed in Table 4.

### 2.7. GC-MS Analysis of Flavor Volatile Compounds

Flavor volatile compounds were extracted according to the method from Bento-Silva et al., with slight modifications [23,24]. Briefly, 4 g of powdered cookie was placed into a 20 mL headspace vial, followed by the addition of 4 mL of saturated NaCl solution and 5–10 μL of 1% cyclohexanone (as an internal standard). The vial was promptly sealed and each sample was prepared in triplicate. The sealed vial was placed in a thermostatic magnetic stirrer at 45 °C for 45 min to equilibrate with stirring. Subsequently, the fiber was exposed to the headspace for 30 min to adsorb volatile compounds. After adsorption, the fiber was retracted, withdrawn from the vial, and immediately inserted into the GC-MS injection port at 250 °C for 5 min of thermal desorption [24].

A DB-5 column (30 m × 0.32 mm × 0.25 μm) was selected to separate the flavor volatile compounds. The carrier gas was helium at a flow rate of 1 mL/min. Without shunting, the temperature program was set to first hold at 30 °C for 5 min, then increased to 80 °C at 5 °C/min, then continued to increase to 160 °C at 3 °C/min, then held for 5 min, then raised to 160 °C at 3 °C/min, and finally increased to 250 °C at 4 °C/min and held for 3 min. The mass spectrometry conditions were set as follows: the interface temperature was 250 °C, the ion source was an EI source at 250 °C, the electron bombardment energy was 70 eV, and the mass scan range was 30–550 *m*/*z*.

Volatile compounds were identified by comparing the mass spectra with those in the Wiley and NIST mass spectral libraries, with a match factor of ≥80 serving as the identification criterion.

### 2.8. Statistical Analysis

The experiments were performed in triplicate, and the data were expressed as mean value ± standard deviation (SD). SPSS 21.0 software was used to calculate means and standard deviations for statistical analysis. Radar charts and principal component analysis (PCA) diagrams were generated by using Origin 2025 software. An independent *t*-test was used to compare UDTP cookies with basic butter cookies for texture and sensory data; *p* < 0.05 was considered as statistical significance.

## 3. Results and Discussion

### 3.1. SEM Images of UDTP

**Figure 1:**
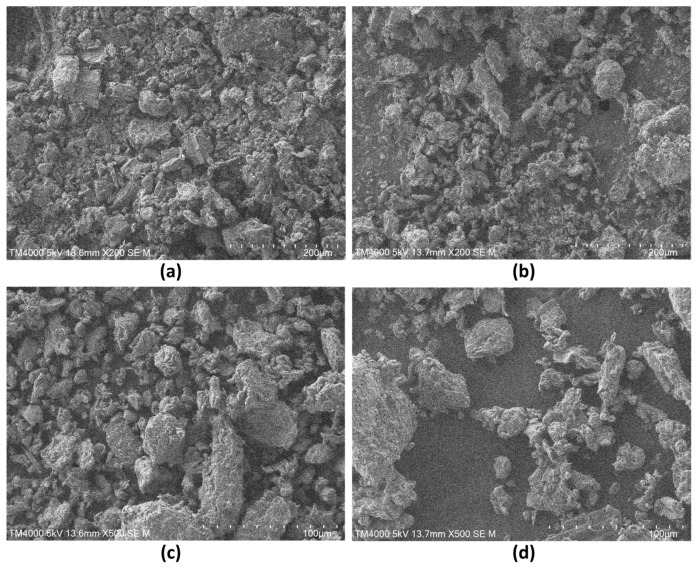
Microscopic appearances of unsieved dark tea powders and UDTP. (**a**,**c**) Unsieved dark tea powder at ×200 and ×500 magnifications, respectively. (**b**,**d**) UDTP at ×200 and ×500 magnifications, respectively.

To characterize the morphological properties of UDTP, the samples were observed via SEM. Significant differences in shape and particle size were observed between unsieved dark tea powder and UDTP (sieved through a 200-mesh sieve). SEM images are presented in Figure 1.

Figure 1a,c show the micromorphology of unsieved dark tea powders at ×200 and ×500 magnifications, respectively. The unsieved dark tea powders exhibited highly irregular shapes, with numerous large and irregular agglomerates formed by the aggregation of fine particles. The surface structure appeared coarse with blurred particle edges, indicating significant clumping or caking. This morphological feature may lead to poor powder flowability and uneven dispersion.

Figure 1b,d present the micromorphology of UDTP at ×200 and ×500 magnifications, respectively. After sieving, the particle uniformity was significantly improved, the particle size distribution was narrowed, and large agglomerates were substantially eliminated. The particles appeared finer, more discrete, and exhibited better dispersion, indicating that the sieving process effectively broke down the original agglomerated structure, yielding a more homogeneous powder.

However, the particle size distribution of UDTP was still not completely uniform. This may be due to two factors: smaller particles accumulate at the bottom and larger particles remain in the upper cavity during grinding, which may hinder the bottom-mounted blades from adequately processing the upper layer; moreover, grinding time is intentionally kept short to prevent overheating, limiting complete pulverization [4]. This residual heterogeneity is consistent with the SEM observations.

### 3.2. Optimization Formulation of UDTP Cookies

#### 3.2.1. Single-Factor Experiment Results

**Figure 2:**
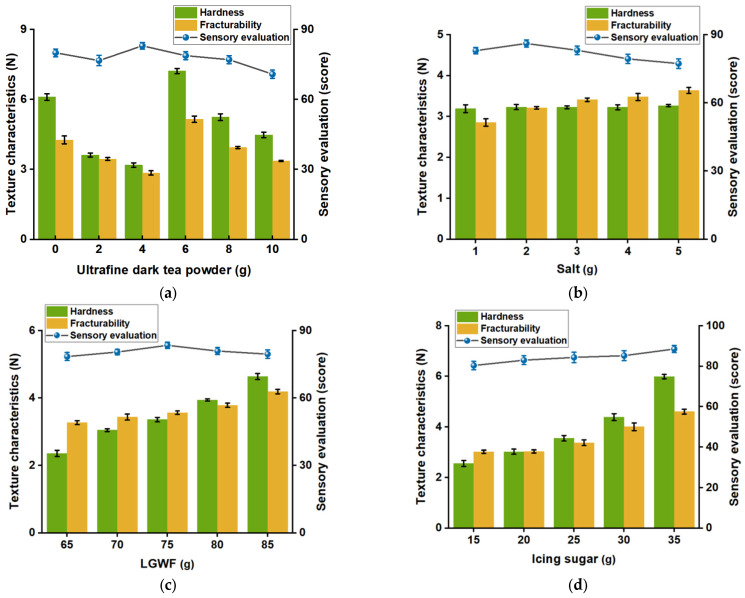
Effect of single-factor experiments on the quality attributes of cookies. (**a**) Effect of UDTP addition level; (**b**) Effect of salt addition level; (**c**) Effect of LGWF addition level; (**d**) Effect of icing sugar addition level.

To evaluate their impact on cookie quality, single-factor experiments were conducted to examine the effects of UDTP, salt, LGWF, and icing sugar, using hardness, fracturability, and sensory score as evaluation indices (Figure 2).

The addition level of UDPT significantly influenced both the textural properties and sensory evaluation of cookies (Figure 2a). Compared to the cookies without UDPT, the hardness and fracturability of UDTP-enriched cookies showed an initial decrease, then increased, and subsequently declined with the increasing UDPT content. In contrast, the sensory evaluation score first increased and then decreased. At a UDPT addition level of 4 g, the cookies exhibited minimum hardness and fracturability, with the highest sensory score of 83 ± 1.41. The reduced hardness and fracturability may be attributed to the biochemical components in the UDTP weakening the binding forces between fat and protein colloids, coupled with its water-holding capacity that effectively inhibits moisture migration [3,25]. However, when the UDTP addition exceeded 4 g, the sensory score gradually declined, and both hardness and fracturability showed a trend of initial increase followed by decrease. This may be because the elevated content of tea polyphenols with increasing UDTP addition inhibits the viscosity of fat, thereby affecting the binding capacity between fats and proteins in the cookies [26]. Additionally, the particle size of the tea powder is also a key factor influencing cookie quality. When the addition of tea powder increases, aggregation and clumping may occur, which could alter the interparticle adsorption and interaction forces and ultimately affect the overall quality of the cookies [27]. Based on these results, the optimal addition level of UDPT was determined to be 4 g.

Salt addition directly affects the flavor profile of food [28]. The influence of salt addition on the cookie characteristics is presented in Figure 2b. As the salt content increased, the hardness of the cookies remained stable, while the fracturability showed a slight upward trend. The sensory score exhibited a pattern of initial increase followed by a decrease with higher salt levels, reaching its peak score of 86 ± 1.56 at an addition of 2 g. When salt addition exceeded 3 g, the excessively salty taste reduced sensory acceptance and visible salt crystals were observed on the fracture surface, resulting in a coarse texture due to increased crystallization. Based on a comprehensive evaluation of textural properties and sensory scores, the optimal salt addition level was determined to be 2 g.

LGWF, as a key component, affects overall fracturability. The effect of LGWF on the sensory attributes and textural properties of the cookies is shown in Figure 2c. The addition level of LGWF significantly influenced the hardness and sensory properties of the cookies. As the amount of LGWF increased, the hardness of the cookies increased significantly, while the fracturability also increased but with a more gradual overall trend. The sensory score exhibited an initial rise followed by a decline, reaching its maximum value at a LGWF addition level of 75 g. Under this condition, the cookies had a moderate mouthfeel, fine and intact structure, uniform tea powder distribution, and no internal air pores. Thus, 75 g was identified as the optimal addition level for LGWF.

Icing sugar is an important raw material for cookies, as it improves dough and product quality by enhancing sweetness and participating in Maillard and caramelization reactions [29]. The effect of icing sugar addition level on the textural properties and sensory evaluation of the cookies is presented in Figure 2d. As icing sugar content increased, both hardness and fracturability showed a continuous upward trend, reaching their maximum values at 35 g addition. The sensory score generally increased gradually. However, at 30 g icing sugar addition, the cookies exhibited a rich flavor, intact appearance, and crisp texture with minimal crumbling. Considering the health implications of excessive sugar intake, and based on a comprehensive evaluation of textural and sensory data, 30 g was selected as the optimal icing sugar addition level.

#### 3.2.2. Orthogonal Test Results

A four-factor, three-level orthogonal experiment was conducted based on the results of the single-factor test (Table 5). According to the range (R) value, the influence of each factor on cookie quality followed the order: A (LGWF) > B (Salt) > D (UDTP) > C (Icing sugar). This indicates that LGWF addition had the most significant influence, followed by salt, UDTP and icing sugar. Based on k-value analysis, the optimal formulation for UDTP cookies was determined to be A_3_B_1_C_1_D_2_. This corresponded to the following addition levels within the base formulation: 80 g LGWF, 1 g salt, 25 g icing sugar, and 4 g UDTP.

### 3.3. Comparative Analysis of Quality Characteristics Between UDTP Cookies and Basic Butter Cookies

Flavor, texture and overall sensory appeal are key determinants of consumer acceptance for cookies [30,31]. Therefore, this study comprehensively evaluated the cookie samples through instrumental texture analysis, sensory evaluation and volatile compound characterization.

#### 3.3.1. Texture Analysis

Texture is a critical quality parameter influencing consumer acceptance of cookies [31]. Hardness and fracturability were measured, and results are presented in Table 6. Compared to basic butter cookies, UDTP cookies showed significantly lower hardness, while fracturability showed no significant difference. Overall, UDTP cookies had a softer texture, which may positively affect consumer acceptability.

#### 3.3.2. Sensory Evaluation

**Figure 3:**
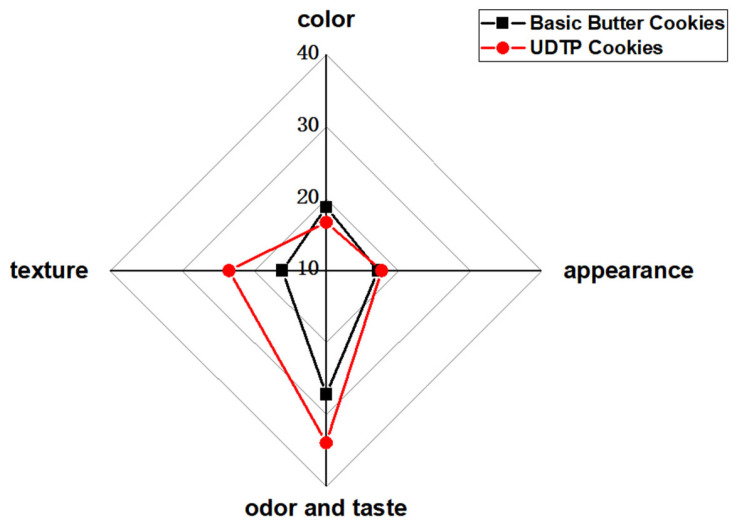
Radar chart of sensory evaluation in terms of color, appearance, odor and taste, and texture. Test was repeated in triplicate.

Sensory evaluation results for color, appearance, odor and taste, and texture are shown in Figure 3. Compared to basic butter cookies, UDTP cookies received higher scores for odor and taste, and texture. However, their color score was lower, as UDTP imparts a deep, greenish-black hue, resulting in a darker appearance than basic butter cookies. No significant difference was observed in appearance scores. Overall, consumers preferred UDTP cookies due to superior odor, taste, and texture.

#### 3.3.3. E-Nose Analysis

**Figure 4:**
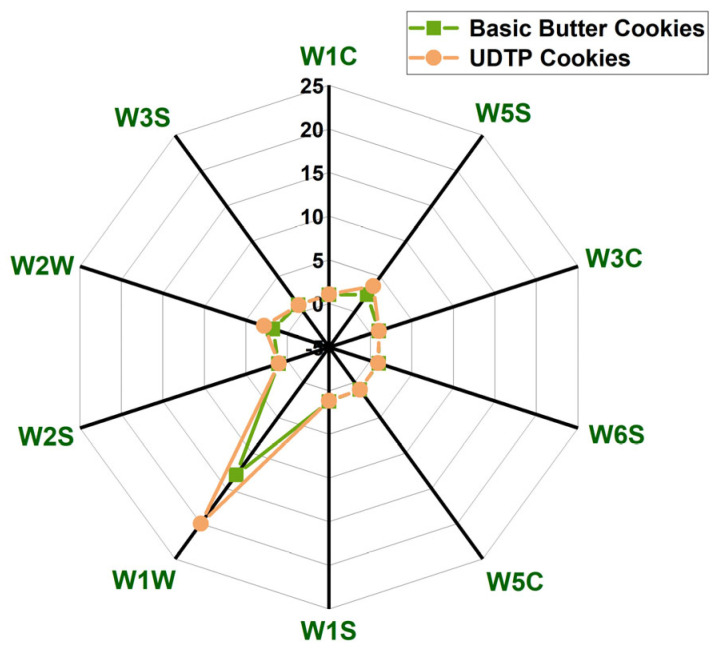
Radar map of volatile components of basic butter cookie and UDTP cookie. The test was repeated six times.

The aroma of cookies is a key determinant of consumer perception and acceptance. E-nose technology rapidly captures a comprehensive volatile profile by monitoring the real-time response of sensor arrays [32,33]. In this study, the 10-sensor array of the E-nose, with each sensor sensitive to different volatile compounds (Table 4), was used to analyze the cookies. A radar chart shows response values for both cookie types (Figure 4).

As illustrated in Figure 4, the radar profiles had similar shapes, indicating broadly comparable volatile profiles. However, differences were observed in the response intensity of specific sensors. Notably, UDTP cookies exhibited significantly stronger responses on three sensors: W1W, W2W, and W5S, with the highest response on the W1W sensor. Responses on the other seven sensors showed no significant variation.

**Figure 5:**
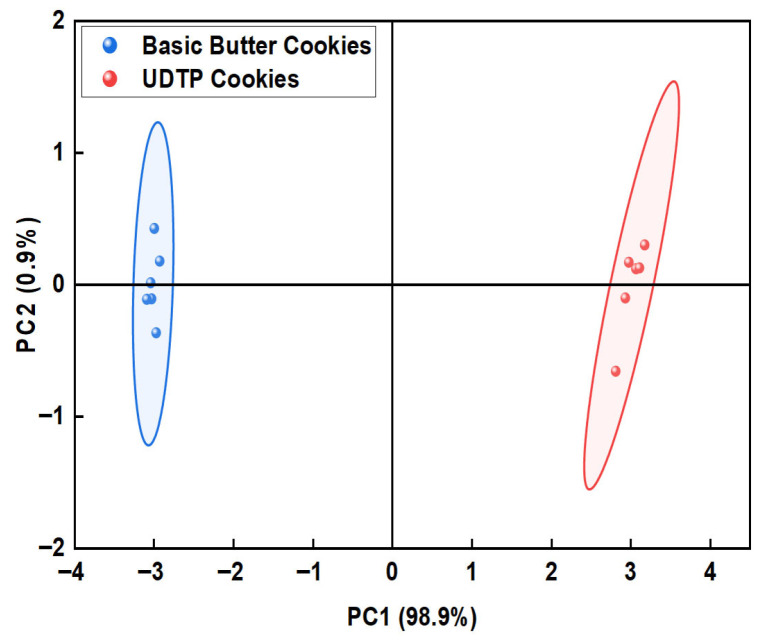
E-nose PCA diagram of basic butter cookies and UDTP cookies. The test was performed six times.

To assess discriminative ability, PCA was applied to sensor response data (Figure 5). The variance contribution rates of PC1 and PC2 were 98.9% and 0.9%, respectively, cumulatively 99.8%. This indicated that PC1 and PC2 effectively reflected the overall sample information. PCA results showed that the samples within the same group clustered together, reflecting high reliability and repeatability of the experiments. Meanwhile, distinct separation between different groups was observed, suggesting that UDTP had a significant impact on the flavor profile of the cookies.

#### 3.3.4. Volatile Compounds

To investigate the influence of UDTP on cookie flavor, GC-MS analysis was conducted to characterize the volatile compounds. As summarized in Table 7, a total of 95 volatile compounds were identified in UDTP cookies, including 12 aldehydes, 12 alcohols, 6 acids, 16 alkanes, 8 ketones, 4 amine, 2 alkenes, 1 aromatic compound, 15 heterocyclic compounds, 4 lactones, 12 esters, and 4 sulfur compounds. Odor descriptions of each compound are provided. Among all the compounds, aldehydes, ketones and heterocyclic compounds were the most abundant, accounting for approximately 15.32%, 12.11% and 34.45% of the total volatile composition, respectively.

Due to their pleasant aroma and low odor thresholds, aldehydes significantly contributed to the flavor profile of the cookies, providing an obvious fatty note [34]. Several desirable aldehydes were identified in UDTP cookies, including hexanal, 2-heptena, nonanal and (E,E)-2,4-decadienal, which are associated with pleasant fat, flower, citrus, and lemon aromas, respectively [35]. Their relative abundances were significantly higher in UDTP cookies than in basic butter cookies. Furthermore, aldehydes such as octanal, (E,E)-2,4-heptadienal and 2-undecena which are associated with pleasant fat and fruit aromas were detected exclusively in UDTP cookies. This is attributed to their role as key constituents of dark tea’s characteristic flavor [36]. Additionally, (Z)-2-nonenal, which exhibits a papar-like odor and originates from the oxidative degradation of linoleic acid, was also found only in the UDTP cookies [37]. Conversely, certain aldehydes associated with off-flavors, such as heptanal, benzaldehyde and (E,E)-2,4-decadienal, which can impart an unpleasant almond note, showed a significant decrease in relative abundance in UDTP cookies.

Ketones, which are primarily formed through the Maillard reaction and lipid oxidation, contribute intense aroma notes even at low concentrations and are commonly associated with fruity and fragrant aromas [38,39]. In UDTP cookies, the relative abundance of 2-tridecanone and 2-undecanone increased significantly, imparting fresh and orange-like scents. Additionally, GC-MS analysis revealed that 1-decen-3-one was detected only in UDTP cookies, likely resulting from the lipid oxidation of tea-specific fatty acids during the baking process.

Alkenes are known to have low odor thresholds, with flower and fruit aromas [34]. Only two alkenes were detected in UDTP cookies: 1-nonene and 3-[(E)-3-methyl-1-butenyl]-1-cyclohexene. 1-nonene was likely formed through the chain cleavage of unsaturated fatty acids present in dark tea during baking. 3-[(E)-3-methyl-1-butenyl]-1-cyclohexene, a monoterpene derivative, was probably generated from terpenoids in dark tea via rearrangement or cyclization reactions under baking conditions.

Pyrazine and furanic compounds are heterocyclic compounds formed primarily through the Maillard reaction, providing a characteristic roasted aroma for baked goods [39]. Compared to basic butter cookies, fewer heterocyclic compounds were detected in the UDTP cookies. This reduction may be attributed to the strong antioxidant capacity of abundant dark tea constituents, such as tea polyphenols, which likely inhibit the formation of these compounds during baking. Despite the overall decrease, certain heterocyclic compounds showed significantly higher levels in UDTP cookies. These included 2,3-dihydro-3,5-dihydroxy-6-methyl-4H-pyran-4-one, 2-furanmethanol, maltol, and 5,5,8a-trimethylhexahydro-2H-chromen-4a(5H)-yl acetate, which provide caramel and fruity aromas. Additionally, 2-ethylpyrazine and 4,6-dimethylpyrimidine were generated, contributing roasted nutty notes. 5-methyl-2-furancarboxaldehyde, furyl hydroxymethyl ketone and 5-hydroxymethylfurfural were also formed, providing roasted and caramel-like aromas.

**Table 7:** GC-MS analysis of volatile components in basic butter cookies and UDTP cookies.

Class	Compound	CAS No./PubChem CID [40]	Molecular Formula	Area (%)		Odor Description [34,41,42,43,44,45,46,47]
				Basic Butter Cookies	UDTP Cookies	
Aldehydes						
1	Hexanal	66-25-1	C6H12O	0.31 ± 0.12	0.64 ± 0.09 *	Fat, apple
2	Heptanal	111-71-7	C7H14O	0.12 ± 0.04	0.21 ± 0.06 *	Greasy smell
3	2-Heptenal	57266-86-1	C7H12O	0.56 ± 0.11	2.86 ± 0.08 *	Grassy, creamy aroma
4	Benzaldehyde	100-52-7	C7H6O	0.64 ± 0.07	0.65 ± 0.09	Burnt sugar, bitter almond
5	Octanal	124-13-0	C8H16O	-	1.53 ± 0.17	Fat, citrus
6	(E,E)-2,4-Heptadienal	4313-03-5	C7H10O	-	0.38 ± 0.11	Fat, nut
7	2-Undecenal	2463-77-6	C11H20O	-	0.18 ± 0.05	Fruit
8	Benzeneacetaldehyde	122-78-1	C8H8O	3.28 ± 0.12	3.06 ± 0.13	Pungent, nut
9	Nonanal	124-19-6	C9H18O	1.53 ± 0.22	3.07 ± 0.10 *	Fat, lemon
10	(E)-2-Dodecenal	20407-84-5	C12H22O	0.28 ± 0.14	-	Fruit
11	(Z)-2-Nonenal	60784-31-8	C9H16O	-	0.71 ± 0.04	Papar
12	10-Octadecenal	56554-92-8	C18H34O	0.10 ± 0.05	-	Fat, oil
13	2,4-dimethyl-benzaldehyde	15764-16-6	C9H10O	0.20 ± 0.02	-	Fruit
14	(E,E)-2,4-Decadienal	25152-84-5	C10H16O	0.48 ± 0.14	1.07 ± 0.09 *	Fat, Oil
15	(Z)-2-Decenal	2497-25-8	C10H18O	-	0.96 ± 0.18	Fat
16	4-Octadecenal	56554-98-4	C18H34O	0.06 ± 0.02	-	Green, citrus
	∑(Aldehydes)			7.56	15.32	
Alcohols						
1	1-Cyclohexene-1-methanol	4845-04-9	C7H12O	-	0.12 ± 0.03	Green
2	1-Octen-3-ol	3391-86-4	C8H16O	0.99 ± 0.02	-	Fat, cucumber
3	5-Octen-2-yn-4-ol	5368947	C8H12O	-	0.06 ± 0.01	Fruit, fat, cucumber
4	(1R,2S,5S)-neodihydrocarveol	18675-33-7	C10H18O	0.08 ± 0.03	0.16 ± 0.09 *	Minty, spearmint
5	2-Decen-1-ol	22104-80-9	C10H20O	0.08 ± 0.01	-	Fruit
6	3-Decyn-2-ol	69668-93-5	C10H18O	0.25 ± 0.04	-	Earthy
7	2-methyl-5-(1-methylethyl)-(1α,2α,5α)-Cyclohexanol	42846-32-2	C10H20O	-	0.31 ± 0.05	Herbal
8	(E)-2-Octen-1-ol	18409-17-1	C8H16O	-	0.28 ± 0.04	Fruit
9	2-(octadecyloxy)-Ethanol	2136-72-3	C20H42O2	0.06 ± 0.03	-	Waxy, fat, oil
10	2-methyl-1-Hexadecanol	2490-48-4	C17H36O	0.04 ± 0.01	0.17 ± 0.05 *	Faint waxy
11	2-Methylene-5α-cholestan-3β-ol	22599-96-8	C28H48O	0.03 ± 0.01	-	NF
12	Octoxynol-5	2315-64-2	C24H42O6	0.07 ± 0.03	-	NF
13	12-Methyl-E,E-2,13-octadecadien-1-ol	90107969	C19H36O	-	0.06 ± 0.02	NF
14	11-Oxa-dispiro [4.0.4.1]undecan-1-ol	558562	C10H16O2	-	0.07 ± 0.04	NF
15	2-(Z)-(9-octadecenyloxy)-Ethanol	5353-25-3	C20H40O2	0.04 ± 0.03	-	NF
16	1-Heptatriacotanol	105794-58-9	C37H76O	0.02 ± 0.01	-	Waxy, oil
17	3,7,11-trimethyl-1-Dodecanol	6750-34-1	C15H32O	-	0.08 ± 0.03	Woody, floral
18	2-Butyl-2,7-octadien-1-ol	5362707	C12H22O	-	0.06 ± 0.01	Green
18	2-Methyl-E,E-3,13-octadecadien-1-ol	5364413	C19H36O	-	0.04 ± 0.02	Waxy, oil, green
19	1-(Cyclopropyl-nitro-methyl)-cyclopentanol	534647	C9H15NO3	-	0.08 ± 0.03	NF
	∑(Alcohols)			1.66	1.49	
Acids						
1	Hexanoic acid	142-62-1	C6H12O2	1.10 ± 0.01	-	Cheese, sour
2	Octanoic acid	124-07-2	C8H16O2	0.54 ± 0.13	0.55 ± 0.09	Cheese, grass
3	Nonanoic acid	112-05-0	C9H18O2	0.46 ± 0.15	0.28 ± 0.13 *	Fat, sour
4	n-Decanoic acid	334-48-5	C10H20O2	0.87 ± 0.23	2.71 ± 0.19 *	Dust, fat, grass
5	trans-13-Octadecenoic acid	693-71-0	C18H34O2	0.02 ± 0.01	-	Waxy, green, oil
6	17-Octadecynoic acid	34450-18-5	C18H32O2	0.02 ± 0.01	-	NF
7	3-hydroxy-Dodecanoic acid	1883-13-2	C12H24O3	-	0.04 ± 0.00	Creamy aroma, Fruit
8	Z-8-Methyl-9-tetradecenoic acid	5364413	C15H28O2	-	0.09 ± 0.03	Waxy, Green, Oil, Fruit
9	Dodecanoic acid	143-07-7	C12H24O2	-	0.10 ± 0.01	Bay laurel
	∑(Acids)			3.01	3.77	
Alkanes						
1	Decane	124-18-5	C10H22	0.63 ± 0.06	0.30 ± 0.11 *	Waxy
2	2,6-dimethyl-Nonane	17302-28-2	C11H24	0.56 ± 0.13	-	Waxy
3	4-methyl-Decane	2847-72-5	C11H24	-	0.44 ± 0.16	Waxy, mineral oil
4	Dodecane	112-40-3	C12H26	0.96 ± 0.23	0.38 ± 0.11 *	Waxy
5	2,6,11-Trimethyldodecane	31295-56-4	C15H32	0.38 ± 0.12	0.74 ± 0.13 *	NF
6	2,6-Dimethylnonane	17302-23-7	C11H24	0.37 ± 0.10	-	NF
7	Undecane	1120-21-4	C11H24	0.18 ± 0.00	0.40 ± 0.12 *	NF
8	2,3-Dimethyldecane	17312-44-6	C12H26	0.09 ± 0.03	0.13 ± 0.01	NF
9	6-methyl-Octadecane	10544-96-4	C19H40	0.26 ± 0.01	0.05 ± 0.00 *	NF
10	2,4-dimethyl-Undecane	17312-80-0	C13H28	0.18 ± 0.06	-	NF
11	2,6-dimethyl-Undecane	17301-23-4	C13H28	0.40 ± 0.13	0.33 ± 0.10	NF
12	4-methyl-Dodecane	6117-97-1	C13H28	0.54 ± 0.22	-	NF
13	3-ethyl-5-(2-ethylbutyl)-Octadecane	55282-12-7	C26H54	0.24 ± 0.03	-	NF
14	2,3,5,8-tetramethyl-Decane	192823-15-7	C14H30	0.19 ± 0.01	0.24 ± 0.05	NF
15	1,1′-[(1,2-Propanediyl)bisoxy]bisoctadecane	35545-51-8	C39H80O2	0.16 ± 0.05	-	NF
16	2,6,10-trimethyl-Tetradecane	14905-56-7	C17H36	0.20 ± 0.09	0.33 ± 0.02	NF
17	Tetradecane	629-59-4	C14H30	0.16 ± 0.03	0.24 ± 0.05	NF
18	Eicosane	112-95-8	C20H42	1.56 ± 0.08	-	NF
19	4-ethyl-Decane	1636-44-8	C12H26	-	0.20 ± 0.03	NF
20	7,7-Diethylheptadecane	91693084	C21H44	-	0.33 ± 0.11	NF
21	2,6,10,14-tetramethyl-Heptadecane	18344-37-1	C21H44	-	0.22 ± 0.05	NF
22	2,6,10,15-tetramethyl-Heptadecane	54833-48-6	C21H44	-	0.14 ± 0.03	NF
23	Hexadecane	544-76-3	C16H34	-	0.40 ± 0.00	NF
	∑(Alkanes)			7.06	4.87	
Ketones						
1	2-Heptanone	110-43-0	C7H14O	1.66 ± 0.13	1.33 ± 0.09 *	Blue cheese, fruit
2	4-Cyclopentene-1,3-dione	930-60-9	C5H4O2	-	0.22 ± 0.07	Caramel, fruit
3	1-(2-carboxy-4,4-dimethylcyclobutenyl)-1-Buten-3-one	5363913	C11H14O3	0.24 ± 0.04	-	NF
4	1,8-diethoxyanthracene-9,10-dione	16294-26-1	C18H16O4	0.42 ± 0.03	-	NF
5	1-Decen-3-one	56606-79-2	C10H18O	-	2.44 ± 0.15	Metallic, rusty
6	3-Nonen-2-one	14309-57-0	C9H16O	0.08 ± 0.05	-	Wet
7	2-Nonanone	821-55-6	C9H18O	3.77 ± 0.13	3.65 ± 0.15	Fragrant, fruit
8	(2R,3R,4aR,5S,8aS)-2-Hydroxy-4a,5-dimethyl-3-(prop-1-en-2-yl)octahydronaphthalen-1(2H)-one	66884-74-0	C15H24O2	-	0.25 ± 0.07	Woody
9	2-Undecanone	112-12-9	C11H22O	2.38 ± 0.13	2.89 ± 0.11 *	Fresh, orange
10	2-Tridecanone	593-08-8	C13H26O	0.60 ± 0.08	1.00 ± 0.03 *	Savory
11	2-Pentadecanone	2345-28-0	C15H30O	0.31 ± 0.12	0.33 ± 0.15	Green
	∑(Ketones)			9.46	12.11	
Amine						
1	4-Acetamidophenol	103-90-2	C8H9NO2	0.64 ± 0.05	0.26 ± 0.02 *	NF
2	2-imino-Cyclopentanecarbonitrile	2321-76-8	C6H8N2	0.46 ± 0.08	-	Unpleasant odor
3	Paromomycin	7542-37-2	C23H45N5O14	-	0.22 ± 0.02	NF
4	2-amino-5-[(2-carboxy)vinyl]-Imidazole	5364104	C6H7N3O2	-	0.06 ± 0.03	Unpleasant odor
5	N-[3-[N-Aziridyl]propylidene]tetrahydrofurfurylamine	537714	C10H18N2O	-	0.07 ± 0.04	NF
	∑(Amine)			1.1	0.61	
Alkenes						
1	1-Nonene	124-11-8	C9H18	-	0.53 ± 0.23	Green
2	3-[(E)-3-Methyl-1-butenyl]-1-cyclohexene	56030-49-0	C11H18	-	0.22 ± 0.09	Green, fresh, orange
	∑(Alkenes)				0.75	
Alkynes						
1	(E)-5-Tetradecen-3-yne	74744-48-2	C14H24	0.04 ± 0.00	-	Green, cucumber
	∑(Alkynes)			0.04		
Aromatic Compounds						
1	Orcinol	504-15-4	C7H8O2	-	0.23 ± 0.04	Woody
	∑(Aromatic Compounds)				0.23	
Heterocyclic Compounds						
1	2-Furanmethanol	98-00-0	C5H6O2	1.75 ± 0.09	3.18 ± 0.19 *	Burnt, caramel, cooked
2	3-Furaldehyde	498-60-2	C5H4O2	-	4.41 ± 0.23	Floral, fruit
3	Furfural	98-01-1	C5H4O2	1.75 ± 0.07	-	Bread, almond
4	2-ethyl-5-methyl furan	1703-52-2	C7H10O	0.49 ± 0.11	-	Gassy
5	1-(2-furanyl)-Ethanone	1192-62-7	C6H6O2	-	0.73 ± 0.08	Balsamic, cocoa, coffee
6	2-Ethylpyrazine	13925-00-3	C6H8N2	-	0.59 ± 0.23	Nut, roasted, rum, wood
7	2,3-Dimethylpyrazine	5910-89-4	C6H8N2	0.07 ± 0.03	-	Caramel, cocoa, hazelnut, peanut butter, roasted
8	4,6-dimethylPyrimidine	1558-17-4	C6H8N2	-	0.29 ± 0.04	Nut, roasted
9	4-vinylcyclohexene dioxide	106-87-6	C8H12O2	0.10 ± 0.01	-	NF
10	5-methyl-2-Furancarboxaldehyde	620-02-0	C6H6O2	NF	0.98 ± 0.02	Almond, caramel
11	2-Propionylfuran	3194-15-8	C7H8O2	0.09 ± 0.02	-	Fruit
12	2-Amylfuran	3777-69-3	C9H14O	0.59 ± 0.13	-	Butter, floral, fruit, green
13	4,4-Ethylenedioxy-pentanenitrile	40159-07-7	C7H11NO2	-	0.19 ± 0.04	NF
14	3-Furancarboxylic acid, methyl ester	13129-23-2	C6H6O3	0.09 ± 0.05	-	Fruit, caramel
15	Furyl hydroxymethyl ketone	17678-19-2	C6H6O3	-	0.36 ± 0.05	Caramel
16	2,3-dihydro-3,5-dihydroxy-6-methyl-4H-Pyran-4-one	28564-83-2	C6H8O4	0.26 ± 0.07	0.98 ± 0.23 *	Caramel
17	4-methyl-1H-Imidazole-5-carboxylic acid	1457-59-6	C5H6N2O2	-	0.47 ± 0.13	NF
18	2H-1b,4-Ethanopentaleno [1,2-b]oxirene, hexahydro-, (1aα,1bβ,4β,4aα,5aα)-(9Cl)	117221-80-4	C10H14O	0.13 ± 0.05	-	NF
19	5-Hydroxymethylfurfural	67-47-0	C6H6O3	-	1.57 ± 0.23	Roasted, caramel
20	dodecyl-Oxirane	3234-28-4	C14H28O	0.06 ± 0.03	0.09 ± 0.01	Aromatic
21	1,2-Epoxyundecane	17322-97-3	C11H22O	-	0.53 ± 0.11	Waxy
22	1b,5,5,6a-Tetramethyl-octahydro-1-oxa-cyclopropa[a]inden-6-one	534400	C13H20O2	0.08 ± 0.01	-	NF
23	Maltol	118-71-8	C6H6O3	5.42 ± 0.23	20.00 ± 0.36 *	Caramel
24	5,5,8a-Trimethylhexahydro-2H-chromen-4a(5H)-yl acetate	54344-83-1	C14H24O3	0.03 ± 0.00	0.08 ± 0.04 *	Fruit
25	Methyl 2-(1-acetyl-5-ethyl-2-[3-(2-hydroxyethyl)-1H-indol-2-yl]-4-pipe ridinyl)propanoate	55724-47-5	C23H32N2O4	0.06 ± 0.03	-	Unpleasant odor
26	(5β)Pregnane-3,20β-diol, 14α,18α-[4-methyl-3-oxo-(1-oxa-4-azabutane-1,4-diyl)]-, diacetate	537242	C28H43NO6	0.04 ± 0.03	-	Unpleasant odor
27	19,21-Epoxy-15,16-dimethoxy-1-acetylaspidospermidin-17-ol	274067854	C23H30N2O5	0.07 ± 0.02	-	NF
	∑(Heterocyclic Compounds)			11.08	34.45	
Lactones						
1	delta-Undecanolactone	710-04-3	C11H20O2	0.32 ± 0.05	-	Peach
2	δ-Tetradecalactone	2721-22-4	C14H26O2	-	0.43 ± 0.08	Dairy
3	γ-Dodecalactone	2305-05-7	C12H22O2	0.02 ± 0.00	0.03 ± 0.03	Apricot, floral, fruit, peach
4	δ-Dodecalactone	713-95-1	C12H22O2	0.13 ± 0.05	0.17 ± 0.06	Fruit
	∑(Lactones)			0.47	0.63	
Esters						
1	Undec-10-ynoic acid, but-3-yn-2-yl ester	91697619	C15H22O2	-	0.10 ± 0.02	Green, cucumber
2	9-Octadecenoic acid (Z)-phenylmethyl ester	55130-16-0	C25H40O2	-	0.04 ± 0.03	Almond, fruit, floral
3	4-Hydroxy-4-methylhex-5-enoic acid, tert.-butyl ester	545523	C11H20O3	0.06 ± 0.04	0.14 ± 0.02 *	NF
4	10-Methyl-8-tetradecen-1-ol acetate	5363228	C17H32O2	0.06 ± 0.01	-	NF
5	Methoxyacetic acid, 4-tridecyl ester	542292	C16H32O3	0.03 ± 0.01	-	NF
6	rel-9-Octadecenoic acid [(2S *)-2α *-phenyl-1,3-dioxolane]-4α *-ylmethyl ester	56599-45-2	C28H44O4	0.03 ± 0.00	-	NF
7	7-Methyl-Z-tetradecen-1-ol acetate	5363222	C17H32O2	-	0.07 ± 0.04	NF
8	E-8-Methyl-9-tetradecen-1-ol acetate	5363273	C17H32O2	-	0.03 ± 0.02	NF
9	Ethyl iso-allocholate	6452096	C26H44O5	0.02 ± 0.01	-	NF
10	Dibutyl phthalate	84-74-2	C16H22O4	0.33 ± 0.05	0.43 ± 0.13	Slight aromatic
11	Hexadecanoic acid, ethyl ester	628-97-7	C18H36O2	0.18 ± 0.07	0.05 ± 0.03	Waxy
12	Linoleic acid ethyl ester	544-35-4	C20H36O2	0.04 ± 0.01	-	Waxy, green, oil
13	(E)-9-Octadecenoic acid ethyl ester	6114-18-7	C20H38O2	0.64 ± 0.05	0.20 ± 0.03 *	Oil, fruit
14	10-Methyl-E-11-tridecen-1-ol propionate	5365029	C17H32O2	-	0.05 ± 0.01	NF
15	Phthalic acid, hex-3-yl isobutyl ester	91719722	C18H26O4	-	0.13 ± 0.05	NF
16	trans-9-Octadecenoic acid, pentyl ester	5462694	C23H44O2	0.05 ± 0.03	-	Waxy, oil
17	Octadecanoic acid, ethyl ester	111-61-5	C20H40O2	0.06 ± 0.02	0.03 ± 0.02	Faint waxy/fatty
18	Butyl 4,7,10,13,16,19-docosahexaenoate	123582940	C26H40O2	0.04 ± 0.00	-	Fishy
19	10,13-Octadecadiynoic acid, methyl ester	18202-24-9	C19H30O2	0.04 ± 0.01	-	NF
20	6,9,12,15-Docosatetraenoic acid, methyl ester	17364-34-0	C23H38O2	0.02 ± 0.01	-	Fishy
21	n-Propyl 5,8,11,14,17-eicosapentaenoate	91697570	C23H36O2	-	0.03 ± 0.00	Fishy
22	Stearic acid, 3-(octadecyloxy)propyl ester	17367-40-7	C39H78O3	0.04 ± 0.02	-	NF
23	3-Cyanotricyclo [4.2.2.02,5]deca-7,9-diene-7,8-dicarboxylic acid dimethyl ester	20185-30-2	C15H15NO4	0.04 ± 0.03	-	NF
	∑(Esters)			1.68	1.3	
Sulfur Compounds						
1	1-Nonanethiol	1455-21-6	C9H20S	-	0.15 ± 0.06	Unpleasant odor
2	Toluene-4-sulfonic acid, 2,7-dioxatricyclo [4.3.1.0(3,8)]dec-10-yl ester	593152	C15H18O5S	0.13 ± 0.04	-	NF
3	tert-Hexadecanethiol	25360-09-2	C16H34S	0.04 ± 0.03	0.06 ± 0.03	Waxy
4	2-Myristynoyl pantetheine	535560	C25H44N2O5S	0.02 ± 0.01	0.05 ± 0.02	NF
5	Desulphosinigrin	5115-81-1	C10H17NO6S	0.04 ± 0.01	-	Bitter
6	3-Methyl-6-(methylthio)hexa-1,5-dien-3-ol	5368118	C8H14OS	-	0.68 ± 0.15	Sulfuraceous, fruit
	∑(Sulfur Compounds)			0.23	0.94	

* *p* < 0.05, compared with basic butter cookies. NF: Not found. The dash (-) represents the aroma compound was not detected. Each volatile compound was expressed as relative percentage of the GC peak area (%) on the total peak areas.

In this study, aldehydes, ketones and heterocyclic compounds were the primary contributors to the flavor profile of cookies. These compounds contributed to the fatty, floral, fruity, fragrant, caramel and pungent aromas. The results indicated that UDTP significantly influenced the flavor composition of the cookies. Specifically, UDTP cookies exhibited a notable increase in abundance of pleasant volatile compounds such as 2-heptenal, octanal, nonanal, 2-undecanone, 2-furanmethanol, 3-furaldehyde and maltol. Consequently, UDTP cookies exhibited richer floral, green, fatty, lemon, fresh, roasted nut, and caramel notes. Overall, the incorporation of UDTP effectively enhanced the flavor profile of cookies by promoting the formation of more pleasant aromatic compounds.

## 4. Conclusions

This study successfully developed a UDTP-enriched cookie through systematic formulation optimization and multi-dimensional quality evaluation. The optimal formulation of UDTP cookies consisted of LGWF 80 g, salt 1 g, icing sugar 25 g, and UDTP 4 g per 80 g butter. UDTP-enriched cookies exhibited significantly lower hardness and comparable fracturability to basic butter cookies, along with higher sensory scores for odor, taste, and texture. GC-MS and E-nose analyses revealed that UDTP enrichment promoted the formation of desirable volatile compounds, particularly aldehydes, ketones, and heterocyclic compounds, contributing to enhanced floral, fruity, roasted nutty, and caramel aromas. These findings demonstrate that UDTP can effectively improve both textural and flavor properties of cookies, offering a viable approach for developing tea-fortified baked products with enriched sensory profiles.

Building on these findings, several avenues for future research are warranted to fully exploit the potential of UDTP in food applications. First, while this study focused on sensory and textural improvements, the functional properties of the cookies remain to be elucidated; therefore, subsequent work should quantify the polyphenol content and antioxidant capacity to substantiate any potential health benefits. Second, given that tea polyphenols are susceptible to oxidation, a systematic evaluation of storage stability, including lipid oxidation indices and the evolution of volatile compounds over time, is essential to establish product shelf-life. Third, to better understand the physical mechanisms behind the improved texture, quantitative particle size distribution analysis should be performed to correlate UDTP granulometry with its functional performance. Fourth, the sensory conclusions could be strengthened by expanding the panel to include a more diverse consumer demographic, and by applying multivariate statistical tools such as PLS-DA to integrate E-nose and GC-MS data, thereby providing deeper insights into the compound-sensory relationships. Finally, the translation of this laboratory-scale formulation to industrial production should be validated through pilot-scale trials, with particular attention to raw material variability, equipment adaptability, and cost efficiency.

In summary, this work demonstrates that UDTP is a promising ingredient for creating baked goods with superior texture and complex flavor profiles. Addressing the outlined future research directions will be critical in advancing UDTP-enriched cookies from a conceptual innovation to a commercially viable functional food.

## Figures and Tables

**Table 1:** Basic formulation (without UDTP) of cookies.

Ingredient	Weight/g	Percentage (on Butter Weight Basis)
Butter	80	100%
LGWF	75	93.75%
Almond powder	30	37.5%
Icing sugar	25	31.25%
Egg yolk	20	25%
Salt	1	1.25%

**Table 2:** Single-factor experiment design.

Factor	UDTP/g	Salt/g	LGWF/g	Icing Sugar/g
1	0, 2, 4, 6, 8, 10	1	75	25
2	4	1, 2, 3, 4, 5	75	25
3	4	2	65, 70, 75, 80, 85	25
4	4	2	75	15, 20, 25, 30, 35

**Table 3:** Orthogonal test design for cookie formulation optimization.

Level	Factor			
	A (LGWF/g)	B (Salt/g)	C (Icing Sugar/g)	D (UDTP/g)
1	70	1	25	2
2	75	2	30	4
3	80	3	35	6

**Table 4:** E-nose sensors and corresponding aroma responses.

Sensor Number	Sensor Name	Sensitive Component
1	W1C	Aromatic compound
2	W5S	Nitrogen oxides compound
3	W3C	Ammonia and aromatic compound
4	W6S	Hydrogen
5	W5C	Alkanes and aromatic compound
6	W1S	Short-chain alkanes (e.g., methane)
7	W1W	Inorganic sulfides, pyrazines, terpenes
8	W2S	Alcohol, aldehyde, ketone, ether
9	W2W	Aromatic components and organic sulfide
10	W3S	Alkanes and long-chain alkanes

**Table 5:** Orthogonal test results for cookie formulation optimization.

No.	Factors				Sensory Evaluation (Score)
	A (LGWF/g)	B (Salt/g)	C (Icing Sugar/g)	D (UDTP/g)	
1	70	1	25	2	81.7 ± 0.91
2	70	2	30	4	78.5 ± 1.91
3	70	3	35	6	77.8 ± 2.22
4	75	1	30	6	79.0 ± 2.48
5	75	2	35	2	78.2 ± 1.57
6	75	3	25	4	78.8 ± 1.36
7	80	1	35	4	84.5 ± 1.21
8	80	2	25	6	81.1 ± 0.83
9	80	3	30	2	81.5 ± 1.15
K1	237.9 ± 1.71	245.2 ± 2.26	241.5 ± 1.26	241.4 ± 1.58	
K2	236 ± 0.32	237.8 ± 1.29	238.9 ± 1.30	241.8 ± 2.79	
K3	247.1 ± 1.54	238 ± 1.56	240.5 ± 3.08	237.8 ± 1.36	
k1	79.31 ± 0.57	81.74 ± 0.75	80.51 ± 0.42	80.46 ± 0.53	
k2	78.67 ± 0.11	79.25 ± 0.43	79.64 ± 0.44	80.59 ± 0.93	
k3	82.36 ± 0.52	79.33 ± 0.52	80.18 ± 1.03	79.28 ± 0.46	
R	3.69 ± 0.41	2.49 ± 0.32	0.87 ± 0.02	1.31 ± 0.48	

**Table 6:** Comparison of textural properties between UDTP cookies and basic butter cookies.

Sample Name	Hardness (N)	Fracturability (N)
Basic Butter Cookies	6.11 ± 0.14	4.27 ± 0.17
UDTP Cookies	4.57 ± 0.08 *	4.18 ± 0.06

* Significantly different from basic butter cookies.

## Data Availability

The original contributions presented in the study are included in the article; further inquiries can be directed to the corresponding authors.

## Abbreviations

The following abbreviations are used in this manuscript:

UDTP	ultrafine dark tea powder
LGWF	low-gluten wheat flour
E-nose	Electronic nose
SEM	Scanning Electron Microscopy
PCA	principal component analysis
GC-MS	gas chromatography-mass spectrometry
